# Velocidade de Onda de Pulso: Chegou a Hora de Reduzir o Ponto de Corte?

**DOI:** 10.36660/abc.20230666

**Published:** 2023-10-26

**Authors:** Marcelo Antônio Oliveira Santos-Veloso

**Affiliations:** 1 Universidade Federal de Pernambuco Programa de Pós-Graduação em Inovação Terapêutica Recife PE Brasil Universidade Federal de Pernambuco – Programa de Pós-Graduação em Inovação Terapêutica, Recife, PE – Brasil; 2 Hospital Alfa Recife PE Brasil Hospital Alfa - Serviço de Clínica Médica, Recife, PE – Brasil

**Keywords:** Hipertensão, Técnicas de Diagnóstico Cardiovascular, Análise de Onda de Pulso

Hipertensão arterial sistêmica (HAS) é a doença crônica não transmissível mais prevalente no Brasil, comumente agravada por distúrbios metabólicos, tais como diabetes mellitus, dislipidemia e obesidade.^1^ Devido ao seu curso silencioso, a pesquisa de lesões subclínicas em estágios iniciais é essencial para instituir medidas de prevenção de complicações cardiovasculares. A identificação de risco com marcadores sensíveis possibilita terapêutica precoce, reduzindo a morbimortalidade associada às doenças cardiovasculares.^2^

A velocidade de onda de pulso (VOP) é considerada o padrão-ouro para identificação de arteriosclerose. Há relação de dose-resposta entre o aumento da VOP e ocorrência de desfechos cardiovasculares maiores, como síndrome coronariana aguda, síndromes cerebrovasculares e morte.^3^

Apesar da importância da VOP, não há consenso sobre o melhor ponto de corte na identificação precoce de lesão de órgão alvo (LOA) em pacientes com HAS. O ponto de corte ideal sofre influência de diversos fatores como método de aferição, etnia, idade, comorbidades e grau de LOA instalado.^4^ As diretrizes europeia, japonesa e coreana recomendam um ponto de corte de 10 m/s para VOP carótido-femoral,^5 - 7^ enquanto a chinesa adota o valor de 12 m/s.^8^

No artigo desta edição, Inuzuka et al.^9^ propuseram-se identificar o melhor ponto de corte de VOP carótido-femoral para identificação de LOA em pacientes HAS na população brasileira. As LOA analisadas foram espessura médio-intimal carotídea (EMIC) ou placa ateromatosa na ultrassonografia de carótidas e hipertrofia ventricular esquerda (HVE) ao ecocardiograma.^9^

Os autores sugerem um ponto de corte de 8,2 m/s, porém com modestos valores de área sob curva (ASC) ROC: 0,678 para EMIC; 0,717 para HVE e 0,649 para placa carotídea. Não foi calculada a curva ROC para o modelo geral, ou seja, no qual a presença de qualquer das três LOAs fosse considerada.

A ASC é um dado amplamente utilizado em estudos diagnóstico e representa a acurácia do método: quanto maior (próximo a 1,0), melhor a capacidade discriminatória entre paciente com e sem LOA. Os valores da ASC podem ser classificados como ruim, moderado, bom ou excelente. Embora não haja consenso, a maioria dos autores classifica como excelente ASC com valores 0,90-1,00; bom entre 0,75-0,90; moderado entre 0,70-0,75 e ruim abaixo de 0,70.^10^

Uma importante limitação do estudo é a ausência do cálculo do intervalo de confiança (IC) 95%. Devido a imprevisibilidade inerente à estatística, o valor real de qualquer variável deve variar 95% das vezes entre dois extremos que chamamos IC. Por exemplo, para uma ASC de 0,70 e IC 95% entre 0,6-0,8 o valor real da ASC não é 0,70, mas sim um valor que pode estar entre 0,60 e 0,80. E, a diferença entre o valor superior e inferior desse intervalo é determinado pelo tamanho da amostra: quanto maior a amostra, menor o intervalo e mais precisa a aferição.

Devido à pequena amostra, é possível que o IC 95% das ASC apresentadas contenha o valor de 0,5 (ex: 0,6 [0,5-0,7]), o que significaria que na verdade não há poder discriminatório do método aplicado.

Recentemente, uma metanálise com dados individuais de 5836 pacientes dos estudos IDCARS e MONICA avaliou o ponto de corte ideal de VOP carótido-femoral para predizer mortalidade geral e eventos cardiovasculares fatais e não fatais. O ponto de corte de 9 m/s foi o melhor para predição dos desfechos, com ASC 0.691 (0.647–0.735) e 0.691 (0.647–0.735), respectivamente, para indivíduos do IDCARS e MONICA.^4^

A despeito das limitações, os resultados de Inuzuka et al.^9^ retratam dados nacionais e convergem com achados mais robustos para indicar a necessidade de redução dos pontos de corte tradicionalmente recomendados pelas diretrizes internacionais.^9^
