# Supplementary material for: Velocidade de Onda de Pulso: Chegou a Hora de Reduzir o Ponto de Corte?
Source: Arq Bras Cardiol. 2023 Oct 26;120(10):e20230666. [Article in Portuguese] doi: 10.36660/abc.20230666 (PMC12080669; doi:10.36660/abc.20230666)
Supplement: Translation

# Translation: Pulse Wave Velocity: Is It Time to Reduce the Cutoff Point?

**Authors:** Marcelo Antônio Oliveira Santos-Veloso

**Affiliations:** 1 Brazil Universidade Federal de Pernambuco – Programa de Pós-Graduação em Inovação Terapêutica, Recife, PE – Brazil; 2 Brasil Hospital Alfa - Serviço de Clínica Médica, Recife, PE – Brasil

**Keywords:** Hypertension, Diagnostic Techniques, Cardiovascular, Pulse Wave Analysis

Systemic arterial hypertension (SAH) is Brazil’s most prevalent chronic non-communicable disease, commonly aggravated by metabolic disorders, such as diabetes mellitus, dyslipidemia, and obesity.^1^Due to its silent course, research into subclinical injuries in the early stages is essential to establish measures to prevent cardiovascular complications. Risk identification with sensitive markers enables early therapy, reducing morbidity and mortality associated with cardiovascular diseases.^2^

Pulse wave velocity (PWV) is the gold standard for identifying arteriosclerosis. There is a dose-response relationship between increased PWV and the occurrence of major cardiovascular outcomes, such as acute coronary syndrome, cerebrovascular syndromes, and death.^3^

Despite the importance of PWV, there is no consensus on the best cutoff point for the early identification of target organ damage (TOD) in patients with SAH. The ideal cutoff point is influenced by several factors such as measurement method, ethnicity, age, comorbidities, and degree of TOD established.^4^European, Japanese, and Korean guidelines recommend a cutoff point of 10 m/s for carotid-femoral PWV.^5 - 7^At the same time, the Chinese guideline adopts the value of 12 m/s.^8^

In the article on this issue, Inuzuka et al.^9^proposed to identify the best carotid-femoral PWV cutoff point for identifying TOD in SAH patients in the Brazilian population. The TOD analyzed were carotid intima-media thickness (CIMT) or atheromatous plaque on carotid ultrasound and left ventricular hypertrophy (LVH) on echocardiography.^9^

The authors suggest a cutoff point of 8.2 m/s but with a modest area under the curve (AUC) ROC values: 0.678 for CIMT, 0.717 for LVH, and 0.649 for carotid plaque. The ROC curve was not calculated for the general model, in which the presence of any of the three TODs was considered.

The AUC is data widely used in diagnostic studies and represents the accuracy of the method: the higher it is (close to 1.0), the better the discriminatory capacity between patients with and without TOD. AUC values can be classified as poor, moderate, good, or excellent. Although there is no consensus, most authors classify AUC as excellent with values 0.90-1.00, good between 0.75-0.90, moderate between 0.70-0.75, and poor below 0.70.^10^

An important limitation of the study is the lack of calculation of the 95% confidence interval (CI). Due to the inherent unpredictability of statistics, the real value of any variable must vary 95% of the time between two extremes that we call CI. For example, for an AUC of 0.70 and a 95% CI between 0.6-0.8, the real AUC value is not 0.70 but rather a value that can be between 0.60 and 0.80. Furthermore, the difference between this range’s upper and lower value is determined by the sample size: the larger the sample, the smaller the range and the more precise the measurement.

Due to the small sample, it is possible that the 95% CI of the AUC presented contains a value of 0.5 (ex: 0.6 [0.5-0.7]), which would mean that there is actually no discriminatory power of the method applied.

Recently, a meta-analysis with individual data from 5836 patients from the IDCARS and MONICA studies evaluated the ideal cutoff point for carotid-femoral PWV to predict overall mortality and fatal and non-fatal cardiovascular events. The cutoff point of 9 m/s was the best for predicting outcomes, with AUC 0.691 (0.647–0.735) and 0.691 (0.647–0.735), respectively, for individuals from IDCARS and MONICA.^4^

Despite the limitations, the results of Inuzuka et al.^9^portray national data and converge with more robust findings to indicate the need to reduce the cutoff points traditionally recommended by international guidelines.^9^
